# The Effect of Clofibrate With Phototherapy on Full-Term Newborns With Non-Hemolytic Jaundice

**DOI:** 10.5812/ircmj.995

**Published:** 2013-04-05

**Authors:** Zohre Torabi, Ali Eskandarzadeh, Akefeh Ahmadiafshar

**Affiliations:** 1 Mousavi Hospital, Zanjan University of Medical Sciences, Zanjan, IR Iran; 2 Mahneshan Hospital, Zanjan University of Medical Science, IR Iran; 3 Zanjan University of Medical Sciences, Zanjan, IR Iran

**Keywords:** Clofibrate, Infant, Newborn, Hyperbilirubinemia, Jaundice

*Dear Editor,*

Hyperbilirubinemia is a common problem in Iranian newborns and constitutes approximately one third of admissions to our neonatal ward. Phototherapy is the most widely used form of therapy for the treatment of neonatal hyperbilirubinemia (1). There are some pharmacological agents introduced for treatment of unconjugated neonatal jaundice, such as Phenobarbital, Metalloporphyrins, and D–penicillaminem but more research is necessary to prove their safety in clinical use (2). Glucuronyl transferase activity can be increased with administration of clofibrate (3). Therefore, this drug has been recently proposed for the treatment of neonatal hyperbilirubinemia (4, 5) but, it has not yet been approved as a routine drug for treatment of neonatal jaundice. The aim of the present study was to determine the effect of 100 mg/kg clofibrate on Total serum bilirubin (TSB) level in healthy term neonates. After receiving written consent from the parents, 100 neonates with hyperbilirobinemia were enrolled in this study. Inclusion criteria were body weight between 2500-3500grams, gestational age between 38 to 41 weeks, breastfed, and having TSB between 17 to 22 mg/dl, postnatal age between 3-7 days. Neonates with any congenital anomaly, hemolytic disease (Rh or ABO incompatibility and a positive combs' test), infection (congenital or acquired), dehydration, G6PD deficiency and conjugated bilirubin more than 1/5mg/dl or exceeding 15% of total serum bilirubin were excluded. In this open labeled study, patients were randomly allocated to the treatment group (clofibrate) or the control group by a simple randomization method using a table of random numbers. All neonates in both groups received phototherapy. Each phototherapy unit contained 4 special white lamps and was adjusted 20 cm above the neonatal cot. The lamps were changed regularly after 250 hours of usage. A single dose of clofibrate (100 mg/kg) was administered orally to infants in the study group within 12 hours of admission. Phototherapy was started immediately on admission for all studied patients until TSB decreased to a safe level (< 14 mg/dl). Total and direct serum bilirubin levels were measured 12, 24, 48 and 72 hours after admission and then it was done daily until phototherapy was discontinued. TSB measurement was performed on the basis of spectrophotometric principles by using Bilimeter 3, Pfaff Medical GmbH, and direct bilirubin measurement, by using Auto-analyzer Random Access (Selectra E, Vital Scientific, Netherlands), respectively. Laboratory tests, including complete blood count, total and direct serum bilirubin, reticulocyte count; and direct Coombs agglutination test, maternal and neonatal blood group, glucose-6-phosphate dehydrogenase determination and peripheral blood smear were performed for all jaundiced infants in both groups. Our criterion for discharge was TSB reduction to a safe level (< 14 mg/dl). All neonates were visited 48 hours and 1,2,3,4 weeks after discharge, with a careful physical examination for side effects of treatment. This study was approved by Ethical Committee of Zanjan University of Medical Sciences. All data were analyzed using statistical package of social sciences (SPSS) software for windows version 14. Statistical analysis of data was performed by independent sample t test and repeated measurement test and P values less than 0.05 were considered significant. One hundred neonates were investigated in this study , the age ,weight ,sex, total bilirubin levels, reticulocyte count, hematocrit and, hemoglobin were similar in clofibrate and control group (P > 0.05). Although bilirubin level reduced in the control and clofibrate groups after 12, 24, 48, and 72 hours, this reduction in clofirate group was statistically significant. It also confirmed by repeated measurement test (P < 0.0001) (Table 1). The duration of phototherapy in Clofibrate group was 24.44 ± 10.13 hours in neonates receiving clofibrate versus 44.4 ± 15.38 in control group (P < 0.0001). On serial examination during hospitalization and between two and four weeks after discharge at the outpatient clinic, no side effects were observed. The neonates in both groups did not need exchange transfusion and hyperbilirulbinemia was controlled by phototherapy. The present study demonstrated that a single dose of clofibrate (100 mg/kg) in healthy full-term breastfed newborn with marked hyperbilirubinemia can significantly reduce indirect bilirubin levels after12 hours of treatment. These findings are consistent with the results of other studies that have demonstrated the efficacy of clofibrate in reduction of indirect bilirubin (5). Our study was carried out in term neonates. However, some studies showed similar results in pre-term and G6PD deficient neonates (4, 5). Clofibrate increases bilirubin conjugation and excretion, faster than phenobarbital and is a better enhancer of glucuronosyl transferas, with no drowsiness (6). Phenobarbital also has a long half-life, causes drowsiness and may increases the risk of bilirubin toxicity in brain (7). Clofibrate has some side effects in adults such as nausea, gastrointestinal disturbance, vomiting and loose stools. None of these side effects were reported in neonates with a single dose of clofibrate (8, 9). We didn’t find any side effects during our study. Indeed, clofibrate only is actually available pharmacologic agent that could be used effectively in neonatal hyperbilirubinemia without any side effects.

**Table 1:** Total Serum Bilirubin Level After 12, 24, 48 and 72 Hours in Clofibrate and Control Groups

Groups	Baseline	12 hr	24 hr	48 hr	72 hr
**Clofibrate, No. (Mean ± SD)**	50 (19.06 ± 0.761)	50 (14.96 ± 0.911)	50 (12.47 ± 1.40)	32 (10.26 ± 1.06)	19 (6.48 ± 1.03)
**Control, No. (Mean ± SD)**	50 (19.17 ± 1.07)	50 (16.58 ± 1.15)	50 (14.57 ± 1.19)	50 (12.55 ± 1.40)	32 (11.23 ± 1.39)
